# Innovative Integration of Dual Quantum Cascade Lasers on Silicon Photonics Platform †

**DOI:** 10.3390/mi15081055

**Published:** 2024-08-22

**Authors:** Dongbo Wang, Harindra Kumar Kannojia, Pierre Jouy, Etienne Giraud, Kaspar Suter, Richard Maulini, David Gachet, Léo Hetier, Geert Van Steenberge, Bart Kuyken

**Affiliations:** 1 Photonics Research Group, Department of Information Technology, Ghent University—imec, 9052 Ghent, Belgium; bart.kuyken@ugent.be; 2 Center for Microsystem Technology (CMST), Ghent University—imec, 9052 Ghent, Belgium; harindra.kannojia@ugent.be (H.K.K.); geert.vansteenberge@ugent.be (G.V.S.); 3 IRsweep AG, Laubisrütistrasse 44, 8712 Stäfa, Switzerland; 4 Alpes Lasers SA, Avenue des Pâquiers 1, 2072 St-Blaise, Switzerland

**Keywords:** silicon photonics, flip-chip integration, quantum cascade laser, mid-infrared spectroscopy

## Abstract

For the first time, we demonstrate the hybrid integration of dual distributed feedback (DFB) quantum cascade lasers (QCLs) on a silicon photonics platform using an innovative 3D self-aligned flip-chip assembly process. The QCL waveguide geometry was predesigned with alignment fiducials, enabling a sub-micron accuracy during assembly. Laser oscillation was observed at the designed wavelength of 7.2 μm, with a threshold current of 170 mA at room temperature under pulsed mode operation. The optical output power after an on-chip beam combiner reached sub-milliwatt levels under stable continuous wave operation at 15 °C. The specific packaging design miniaturized the entire light source by a factor of 100 compared with traditional free-space dual lasers module. Divergence values of 2.88 mrad along the horizontal axis and 1.84 mrad along the vertical axis were measured after packaging. Promisingly, adhering to i-line lithography and reducing the reliance on high-end flip-chip tools significantly lowers the cost per chip. This approach opens new avenues for QCL integration on silicon photonic chips, with significant implications for portable mid-infrared spectroscopy devices.

## 1. Introduction

Mid-infrared spectroscopy is a highly discriminatory technique used for chemical analysis of gases, liquids and solids. In particular, the spectral region (1350–1410 cm^−1^) covers the fundamental vibrational transitions of CH_3_, the basis for analysis of aliphatic hydrocarbons groups [1]. Spectroscopic techniques allow non-destructive and label-free quantitation of a wide variety of samples [2], for instance, oil-in-water, which is crucial for industrial waste evaluation, environment protection and drinking water analysis. Quantum Cascade Lasers (QCLs) are ideal light sources due to their flexible energy bandgap engineering allowing one to design and make lasers with almost any specific emission spectrum in the MIR region from 4 μm to >10 μm, as well as in the THz region [3]. Conventionally, absorption spectroscopy can employ two collimated QCLs as one for measuring traces of analyte while the other for simultaneous background reference, and combines them with free-space beam combiner. However, this free-space routing leads to a bulky setup which is prone to mechanical disturbances and requires expensive optical and opto-mechanical components. The integration of QCLs on photonic integrated circuits (PIC) tends to be an appealing solution, allowing for robustness as there are no moving parts, as well as a reduced fingerprint of the device, and the potential of low-cost production.

Silicon photonics (SiPh) has evolved at a rapid rate by leveraging the extremely mature silicon manufacturing ecosystem as a result of decades of CMOS development [4]. A diverse portfolio of photonic sensors can potentially be integrated on a single silicon chip. In recent years, there has been a growing number of demonstrations of integrated long-wavelength silicon devices, including lasers [5,6], resonators [7,8], modulators [9,10], couplers [11], and multiplexers [12]. Among these components, optical amplifiers have proven to be the most challenging due to their distinct material system and optical interface to passive circuits. Monolithic integration tends to be an ultimate approach where a few demonstrators appeared [13,14,15,16]. However, specific process developments are always required, such as either regrowth or ion implantation. According to the SiPh roadmap of 2024 [17], both heterogeneous and monolithic integration are still at a lower technology readiness level (TRL). By contrast, hybrid integration is appealing as a rapid solution due to its mature technology developed in the near-infrared range [18,19,20]. With regard to the integration of power-hungry QCLs, which requires high thermal conductivity between lasers and silicon substrate, solder-based flip-chip bonding has intrinsic higher thermal conductivity than adhesive bonding which is commonly used in the other approaches.

Germanium (Ge) has low losses in the 2–14 μm wavelength range [21,22,23], which together with its compatibility with standard CMOS processes, makes it the platform of choice for the implementation of mid-infrared PICs. For example, a functional passive circuit sensor over the 6.5–7.5 μm range has been demonstrated based on Ge-on-Si waveguides from our group [24]. As we are now placing efforts on the integration of QCLs on this platform, we reported our first QCL integration results during the IEEE SiPh conference 2023 [25]. Although the initial laser could work only under pulsed operation, it was still very promising by showing the proof-of-concept of 3D self-alignment assembly.

**Figure 1:**
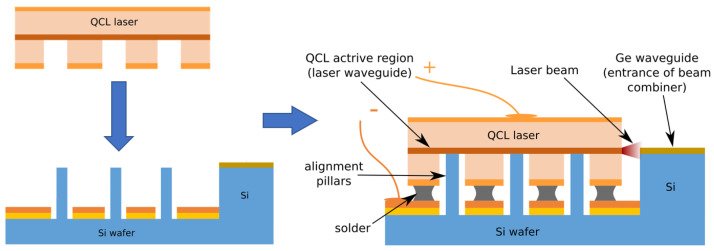
Schematic representation of the 3D self-alignment flip-chip assembly.

In this paper, we elaborate more details on the design, fabrication, assembly, characterization and package of integrated beam combined QCL lasers. The opto-mechanical interface between the Ge-on-Si chip and the InP-based DFB QCL was carefully co-designed to maximize the coupling efficiency between the QCL’s and Ge’s waveguides. Figure 1 shows the 3D self-alignment approach implemented in this paper. The in-plane alignment can be realized by coarse aligning with the lithography markers and vertical alignment can be ensured by mechanical stopper, similarly to Ref. [26]. The bonding was followed by a reflow process to melt the solder which provides capillary force for 3D self-alignment. The optimized solder printing process leads to continuous wave (CW) operational lasing with sub-milliwatt output power. Furthermore, we packaged the beam combined chip in a high heat load (HHL) package that included a thermoelectrical cooler (TEC) and a collimating lens. Both optical power and beam quality were characterized after packaging, resulting in the successful miniaturization of the entire light source module.

## 2. Ge-on-Si PIC

### 2.1. Interface Simulation

**Figure 2:**
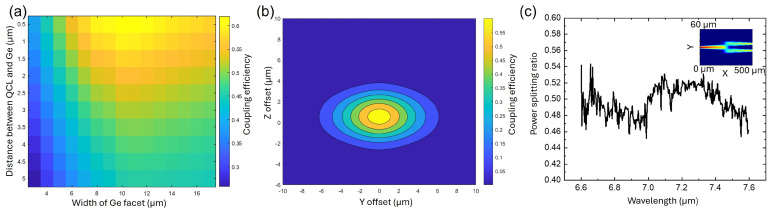
(**a**) Simulation of coupling efficiency at different Ge width and gaps, (**b**) Misalignment simulation, (**c**) Experimental measurement result of 1 × 2 MMI combiner/splitter, insertion shows the simulated top-view optical profile at 7.2 μm wavelength.

To achieve optimal coupling efficiency, the optical mode overlap between the QCL facet and the Ge waveguide facet was simulated by using Ansys, Inc. Lumerical FDTD, Irvine, CA, USA. A Ge thickness of 2 μm was selected to ensure consistency with our platform development [24]. Typically, a spot size converter with an inverse taper is employed to efficiently couple light from III–V to Si/Si_3_N_4_ waveguides in the telecom range. However, this approach requires waveguide features to be a few hundred nanometers in size. Since we aim to adhere to i-line contact lithography processes to significantly reduce costs, we used an adiabatic taper to expand the mode field for maximum matching with the mode in the QCL. Figure 2a presents the simulated coupling efficiency for various widths of the Ge waveguide. The highest coupling efficiency, 61.8%, was achieved using a 10 μm wide Ge taper with no gap. Notably, the coupling efficiency is relatively insensitive to the longitudinal gaps due to the long wavelength; even a 2 μm gap can still provide a reasonable coupling efficiency of 55%.

Figure 2b illustrates the impact of misalignment in both the in-plane (Y) and vertical (Z) directions. There is an inherent trade-off between achieving higher coupling efficiency and ensuring larger alignment tolerance. While a 10 μm width for the Ge waveguide offers the highest coupling efficiency, a 15 μm width provides improved tolerance to misalignment. Therefore, we incorporated both in the experimental assembly. Along the vertical direction, those pedestals sticking out of Si cavity will physically support the QCL. The pillars on the Si PIC were designed to accommodate the geometry of the QCL, with dimensions slightly smaller by ±15 μm laterally and ±20 μm along the cavity to account for uncertainties in QCL cleaving. Due to the high aspect ratio of the laser (several millimeters in length and hundreds of microns in width), significant rotational misalignment is not anticipated.

Single TM mode waveguide with a width of 3.6 μm was employed to direct light to a precisely designed on-chip beam combiner/splitter. A multimode interference (MMI) coupler was selected for the target wavelength of 7.2 μm, owing to its inherent advantage of broad bandwidth. The optimized interference region is 20 μm wide and 110 μm long. As depicted in Figure 2c, the power splitting ratio varies by less than 10% over the wavelength range from 6.6 μm to 7.6 μm. This stability is highly advantageous for dual-comb spectroscopy, wherein two QCL comb lasers with slightly different repetition rates must be combined and directed to a photodetector [27]. A bend radius of 300 μm was implemented throughout the entire circuit, resulting in negligible bending loss.

### 2.2. Process Flow and Fabrication

**Figure 3:**
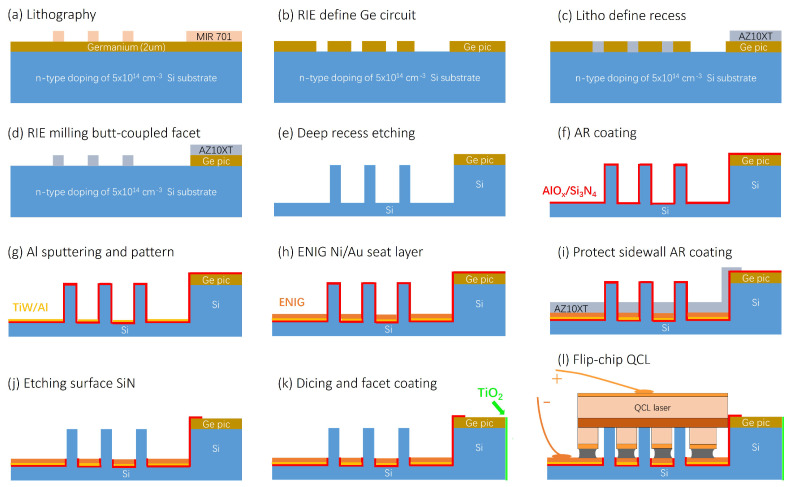
Process flow for self-assembling QCL on Ge-on-Si PIC.

The fabrication process flow for Ge-on-Si PIC followed by flip-chip integration of the DFB QCLs is presented schematically in Figure 3. At first, 2 μm thick Ge layer was epitaxially grown on a 8-inch Si wafer. On top of the Ge layer, high-resolution positive tone MIR701 photoresist was spin-coated and patterned to form waveguide structures using a standard i-line lithography process (Figure 3a). Owing to prior optimizations, MIR701 allowed a minimum feature size of 800 nm with the most smooth sidewall by reflowing it at 110 °C for 1 min before development. The patterned MIR701 was used as a soft mask to etch Ge layer and fabricate Ge waveguides on silicon using reactive ion etching (RIE) with a mixture of CF_4_, SF_6_ and H_2_ (Figure 3b). Afterwards, the remaining resist mask was stripped by rinsing in acetone, IPA and DI water followed by oxygen plasma. The next step was to fabricate the recess and Si pedestals in the Si wafer where the QCLs would be resting to achieve vertical alignment of the QCL’s active region with the Ge waveguide. The Si pedestal and recess design was first patterned in thick AZ10XT photoresist (Figure 3c) which acted as masking layer for deep Si RIE step. However, to achieve an uniform coupling facet in Ge-on-Si PIC, AZ10XT was designed in such a way that an additional RIE step was required to remove Ge on top of the Si pedestals and simultaneously define the coupling facet as well, as shown in Figure 3d. Finally, deep RIE was used to define 20 ± 1 μm deep recess in Si wafer (Figure 3e).

Subsequently, a 40 nm thick AlO_*x*_ layer was deposited using electron beam evaporation to act as an etching stopper due to its high etch selectivity, followed by an anti-reflection (AR) Si_3_N_4_ layer deposition using a conformal PECVD deposition technique (Figure 3f). This was followed by sputter deposition of a TiW adhesion layer (25 nm) and a 350 nm thick Al seed layer which were patterned using standard lithography and wet etching techniques (Figure 3g). Here, H_3_PO_4_/HNO_3_/HAc was used to remove the Al layer, followed by H_2_O_2_ to remove the TiW layer, ensuring that only the conductive pattern remains for the next step. Then, a standard Electroless Nickel Immersion Gold (ENIG) plating was conducted to achieve 3 ± 1 μm thick ENIG pads (Figure 3h) inside the Si recess as well as on the Si surface for wire bonding and further characterizations. The top layers of Si_3_N_4_ and AlO_*x*_ were then removed, resulting into the presence of these materials only on the facets (Figure 3i,j). A combination of RIE dry and BOE wet etching was utilized to ensure a smooth waveguide surface. A 990 nm thick TiO_2_ layer was deposited by electron beam evaporation directionally on the output facet to maximize the optical transmission (Figure 3k). After completing these steps, the chip was diced into separate dies ready for assembly. Finally, the 3D self-alignment flip-chip integration was performed (Figure 3l) which will be discussed in the subsequent section.

**Figure 4:**
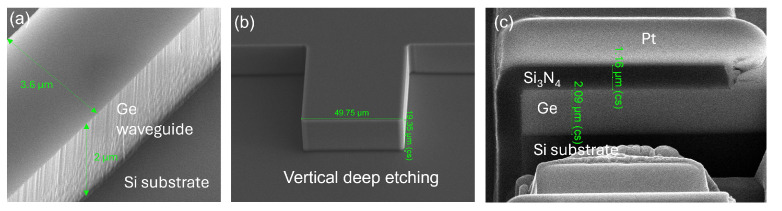
SEM images of (**a**) single mode Ge waveguide, (**b**) vertical 20 μm deep etched Si recess, (**c**) Si_3_N_4_ AR coating on coupling facet.

The fabricated Ge-on-Si PIC was inspected in a scanning electron microscope (SEM) to characterize the waveguide and coupling facet quality. SEM micrograph presented in Figure 4a shows the low sidewall roughness achieved using i-line contact lithography. Despite this, a propagation loss of approximately 7.5 dB/cm was measured for the wavelength of 7.2 μm, implying that a significant portion of the loss originates from the interface between Si and Ge. IMEC Leuven, Belgium has developed a new technology named Ge-on-Nothing for solar cells application, which shows promise for use here as well, effectively avoiding the interfacial defects [28]. Vertical etching of the cavity, as illustrated in Figure 4b, is essential to avoid a positive angle, which would hinder the QCL from moving forward to the Ge facet. Since an uncoated Ge surface reflects 36% of light in the mid-infrared wavelength range, an effective anti-reflection (AR) coating is crucial to reduce reflections. From Figure 4c, a uniform Si_3_N_4_ deposition can be seen in a conformal manner, ensuring thorough coverage of the facet. The optimal thickness for minimal reflection at the target wavelength was determined through both FDTD simulation and experimentation, with 1.117 μm of Si_3_N_4_ reducing reflection down to 1.2% at the target wavelength.

### 2.3. Optical Inspection

**Figure 5:**
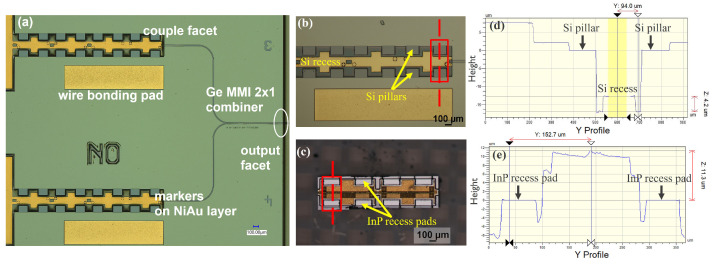
Microscope images of Ge-on-Si PIC (**a**,**b**) and InP-based QCL (**c**), step height profile of Ge-on-Si PIC with ~4 μm thick ENIG layer (**d**) and QCL (**e**).

The optical image of the fabricated Ge-on-Si PIC is shown in Figure 5a. The PIC was designed to have versatile geometry which could accommodate QCLs having different lengths (i.e., 1.5 mm, 2.25 mm, and 3.0 mm), corresponding to three large rectangular QCL building blocks intentionally designed to prevent solder pile-up at the rear of the QCL during assembly. Additionally, the ENIG metal layer inside the cavity was specifically designed with a forward offset of 20 μm with respect to the pillars, providing a capillary pulling force during the solder reflow step for the self-alignment of QCL with the Ge facet along the longitudinal direction. The symmetric shape across the x-axis ensures that the molten solder will center the QCL, minimizing lateral misalignment. Dedicated alignment markers were also incorporated in the ENIG layer to facilitate the coarse alignment during the assembly process.

The beam combining chips consist of one Ge-on-Si PIC and two DFB QCLs specially co-designed by Alpes Lasers, Saint-Blaise, Switzerland. The DFB QCLs are based on a strain-balanced In_0.586_Ga_0.414_As/Al_0.566_In_0.434_As active region similar to Ref. [3]. The active region and a 200 nm thick lattice-matched InGaAs grating host layer were grown by molecular beam epitaxy on an InP substrate. Then, the epiwafer was processed in buried-grating, buried-heterostructure lasers as described in Ref. [29]. All chips were inspected prior to assembly. As shown in Figure 5b–e, the Si pillars on the Ge-on-Si chip and the corresponding InP recess pads were flat and free from any residues or unwanted particles. The QCLs were flipped epitaxy side down and aligned with the dedicated alignment markers on the ENIG layer on the Ge-on-Si PIC such that the QCLs fit perfectly into the Si recesses, with the InP recess pads resting on the Si pillars. Additionally, during the solder reflow process, the self-alignment mechanism pertaining to the capillary forces due to the design offset, as described before, would automatically position the QCL along the x-axis symmetrically and pull it forward until it meets the mechanical stopper. Vertically, the Si pillars ensure sub-micron accuracy, which is determined by the CMOS process. Therefore, the final alignment accuracy is primarily determined by the geometry of the predesigned Ge-on-Si PIC and QCLs, rather than by the precision of flip-chip tools. This allows for the use of lower-cost, downgraded tools, thereby reducing overall costs.

## 3. Assembly

**Figure 6:**
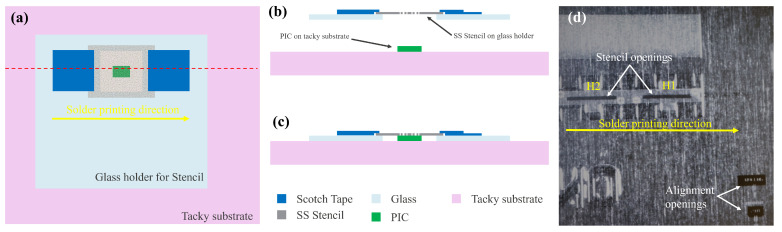
Stencil alignment step using Tresky’s micro-assembly tool. (**a**) Top view of stencil aligned on PIC on tacky substrate, (**b**,**c**) cross-sectional view before and after alignment, and (**d**) split-view image of aligned stencil with Ge-on-Si PIC.

For the assembly of DFB QCLs on the Ge-on-Si PIC, the first step was to deposit solder paste on the ENIG metal pads in the Si recess. A screen printing technique was selected for this owing to its simplicity and availability of the suitable picosecond laser to fabricate stencils with high-quality edges and repeatability. The basic idea was to fabricate stencil in thin stainless steel (SS) foils and mount it on a 2-inch glass substrate having a larger opening at the pre-defined location to facilitate stencil pick-up and placement during alignment step using Tresky’s micro-assembly tool and solder printing. The glass substrate thickness was specifically selected to be the same as that of the PIC so that when the stencil is aligned and placed on top of the PIC, the stencil mounted on top of the glass substrate touches the PIC’s top surface which is in the pre-defined glass opening region, as shown schematically in Figure 6a–c. In this work, the stencils were fabricated from 25 μm thick stainless steel (SS) foils using a 532 nm picosecond laser having an estimated spot size of 30 μm. The dimensions of the printed solder patches were measured as a function of different stencil opening shapes and dimensions to ensure sufficient solder volume was screen printed in the Si recess. Upon optimization, a rectangular stencil opening of 40 × 500 μm^2^ was finalized which resulted in the target solder imprint area of 150 × 400 μm^2^ on the ENIG layer in the PIC’s Si recess. The maximum height of the solder bump before flip-chip bonding was observed to be 45 ± 5 μm, which reduced to a maximum height of 4 ± 2 μm after the solder reflow process. The maximum height of the solder bumps before flip-chip bonding was designed to be higher since the flux will evaporate and the solder will also flow during the solder reflow process to cover the entire ENIG pad.

**Figure 7:**
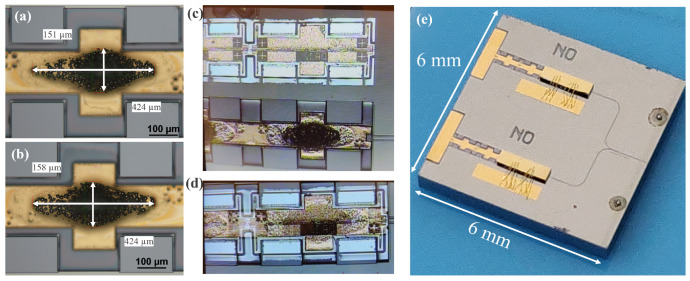
(**a**,**b**) Solder imprint area, (**c**,**d**) Split-view camera images, (**e**) Two QCLs integrated on a single Ge-on-Si PIC.

Therefore, at first, the fabricated stencil was mounted on a glass substrate having a pre-defined opening using single-sided adhesive tape on two sides (as shown by blue patches in Figure 6a). On the other hand, the PIC was placed on a mildly tacky substrate. Thereafter, the stencil attached to the glass substrate was picked up and aligned using a split-view camera of the Tresky’s micro-assembly tool. An actual fabricated SS stencil aligned on the PIC during the alignment process is shown in Figure 6d (whose cross-sectional view is presented schematically in Figure 6b). Once the PIC and stencil were perfectly aligned, the stencil was lowered to rest on the tacky substrate (Figure 6c). The tacky substrate prevented any relative movements between the PIC and stencil, ensuring that they remained aligned until the solder printing was completed. Solder printing was always performed along the longitudinal direction of the laser, i.e., schematically shown as horizontal direction in Figure 6a,d. The solder used in this work was low melting point alloy (LMPA) type-06 solder paste (supplied by Interflux Electronics, Ghent, Belgium). The solder imprint area for two different locations on the same PIC corresponding to the optimized stencil opening before the flip-chip bonding is presented in Figure 7a,b. The flip-chip assembly was performed using Finetech micro-assembly tool. The QCLs were picked up using a vacuum nozzle and then aligned with each other using a split-view camera, as shown in Figure 7c,d. Finally, the aligned QCL was placed onto the PIC.

**Figure 8:**
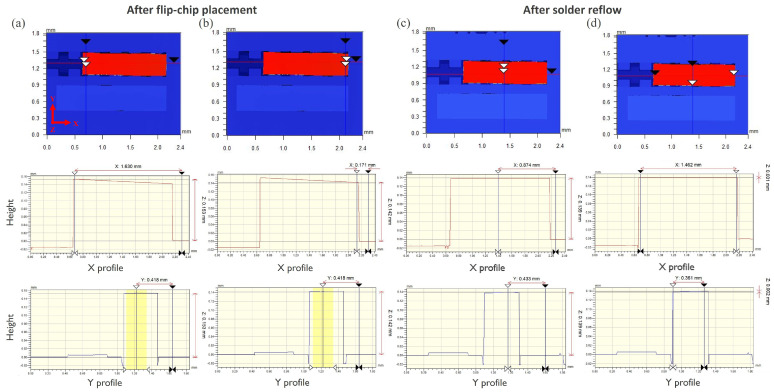
QCL height on Ge-on-Si PIC (**a**,**b**) after flip-chip placement, (**c**,**d**) after solder reflow.

The samples were then reflowed in an oven using a standard thermal program with a maximum temperature of 250 °C. The QCLs were inspected for their step height on the PIC before and after the solder reflow step (Figure 8) to verify that the QCL have landed correctly on the PIC’s recesses and to observe if there was any QCL movement during the reflow step which would facilitate the QCL’s solder-based self-alignment. The step-heights were measured by a non-contact optical profilometer. The step height of the QCL was observed to be 142 ± 2 μm at the location (Figure 8b) where the bonding force was applied, which was in accordance with the expected values considering the Si recess depth and solder bump height. However, the other side of the QCL had a step height of 153 ± 2 μm as shown in Figure 8a. The mismatch in the QCL’s step height on the left and right-side solder bumps can be attributed to the fact that QCL was picked up from the right side rather than from the center. The practice of picking up the QCL from one side not only avoids cracks but is also compatible with different cavity lengths without the need for customized nozzles. But this indeed resulted in a higher direct force application on the right solder bump and a smaller indirect force on the left solder bump. However, this angular misalignment of the QCL in the XZ plane was observed to be corrected by the solder-based self-alignment mechanism during the solder reflow step, as shown in Figure 8c,d. After the solder reflow step, the QCL exhibited a uniform step height of 139 ± 2 μm in both the X- and Y-directions. Given the initial step height of the QCL measured at 150 ± 6 μm and the Si recess with the ENIG layer measured at 12 ± 2 μm, the post-reflow step height aligns with the expected values. This indicates that the QCL’s InP recess landed correctly on the Si pillars of the PIC.

The final step was wire bonding from QCL’s back contact to the ENIG bond pad on Ge-on-Si PIC to provide electrical connections. Figure 7e showcases the successful flip-chip assembly of two QCLs onto Ge-on-Si PIC, with six connections of ø18 μm gold wires, resulting in a compact size of 6 mm × 6 mm. Before packaging, characterization was performed, and both lasers delivered sub-milliwatt optical output power, which will be discussed in the next section.

## 4. P-I-V Characterization

### 4.1. Pulsed Operation

**Figure 9:**
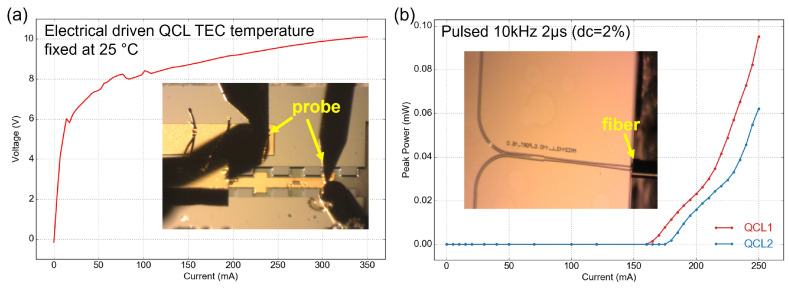
(**a**) I-V curve at room temperature, (**b**) optical power under different injection current, insertions show the microscope images during measurement.

A dedicated setup was constructed to characterize the integrated QCLs using a pulsed current source. The setup comprises probes, cameras, and a single-mode (SM) chalcogenide AsSe fiber to collect light from the chip’s output facet, as depicted in Figure 9 insertions. The emitted light was directed through the fiber onto an HgCdTe (MCT) photovoltaic detector (Vigo) to generate a photovoltage signal. This photovoltage signal was then synchronized with the trigger signal from the pulsed current source and amplified using a microwave lock-in amplifier. Figure 9a shows a representative I-V curve under pulsed operation, indicating that the soldered QCLs and wire bonding process exhibited proper electrical properties. Figure 9b presents the peak power at different injection currents for bonded QCLs, both of which delivered similar output power. It is worth noting that the peak power shown here is calibrated based on the lock-in readings. It includes all setup-induced losses, such as chip-to-fiber coupling loss, fiber propagation loss, and fiber-to-detector coupling loss. Therefore, the actual peak power under pulsed operation could be at least 10 dB higher than the measured value. The threshold current of the bonded lasers was around 170 mA, which corresponds well with the expected values before bonding. The discrepancy between two lasers is small, which could be attributed to the native properties of the QCL chips.

### 4.2. CW Operation

**Figure 10:**
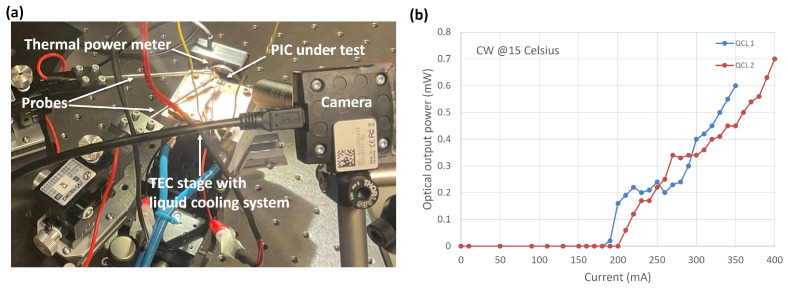
(**a**) CW operation characterization setup. (**b**) P-I curve.

Figure 10a shows the experimental setup for CW characterisation. The PIC, featuring two flip-chip bonded QCLs, was placed on a temperature-controlled stage equipped with both a thermoelectric cooler (TEC) and an anti-freeze liquid cooling system. A thermal power meter (THORLABS S401C) was directly positioned at the front output facet of the PIC to measure the optical power. An estimated 90 % collection efficiency could be expected due to the beam diffraction. Figure 10b illustrates the optical power curves obtained from the two QCLs at 15 °C. Notably, the threshold current in CW operation is slightly higher compared to pulsed mode because of heat accumulation. Both lasers delivered nearly 0.7 mW output power, making them suitable for spectroscopy applications. It is worth noting that all the lasers demonstrated here have a length of 1.5 mm. Our assembly approach is compatible with different lengths up to 3 mm, allowing for higher output power if needed.

## 5. Packaging

**Figure 11:**
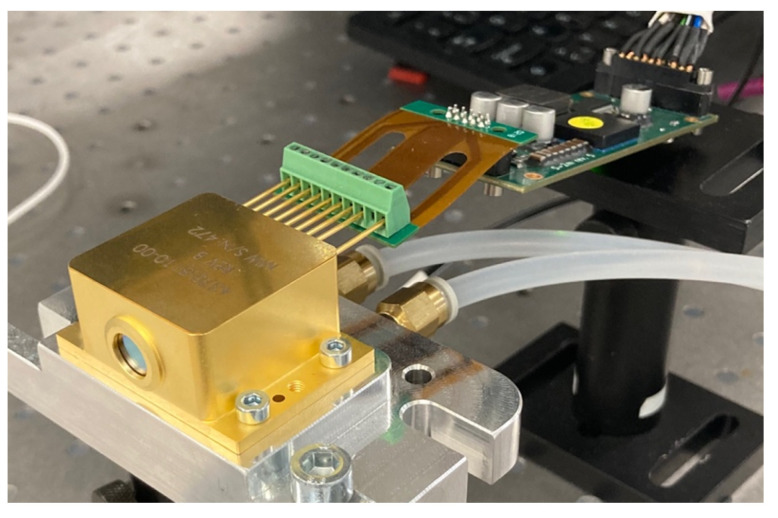
Fully packaged PIC in an HHL module connected to the S2-M driver.

Packaging is a critical final step to miniaturize the entire light module. The beam combining chip was soldered on a submount and packaged in a high heat load (HHL) package, which includes a TEC for temperature control and a beam-collimating lens, and features a sealed design for operation in harsh environments. An antireflective-coated infrared-transparent ZnSe window allows the beam to exit the package with minimal reflective losses. The final package, together with an Alpes Lasers S2-M driver, which controls the applied bias and the TEC’s temperature can be seen on Figure 11. The HHL module is placed on a water-cooled cooling plate to enhance the TEC performance.

Alpes Lasers’ usual HHL packaging process of single QCL sources (see https://www.alpeslasers.ch/encapsulation-in-hhl-housing/ (accessed on 4 January 2024)) was adapted to accommodate the beam combining chip inside the package. Specific solder was chosen to solder the beam combining chip on the submount. The wire-bonding and pinning were adapted to allow current driving of the two QCLs on the beam combining chip.

**Figure 12:**
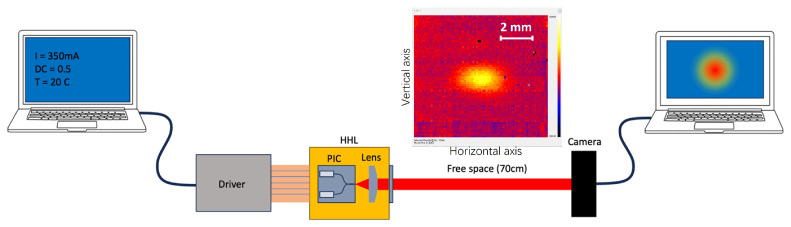
Lens alignment setup and beam shape after 70 cm of free-space propagation.

Beam characterization at the output of the HHL package was performed using the setup depicted in Figure 12. A computer controlled the S2-M driver and collected data. The beam’s position and shape were measured using a camera set 70 cm from the laser. A beam measurement acquired with this test bench is shown in Figure 12 as well. From this beam image, the beam divergence at the HHL output can be extracted. Divergence values of 2.88 mrad along the horizontal axis and 1.84 mrad along the vertical axis were measured. The measured PI curve shows some discrepancies compared to the measurements taken before packaging. Firstly, the lasing threshold increased to 250 mA, which can be attributed to laser degradation. Secondly, the maximum collected optical power reached 0.3 mW at an injected current of 0.35 A and a temperature of 15 °C. Ongoing work aims to further investigate and improve the PIC performance.

## 6. Conclusions

In this paper, we demonstrated a novel approach in the integration of QCLs on Ge-on-Si photonic chip, leveraging a 3D self-alignment flip-chip assembly method. The integration of QCLs on PICs offers a robust, miniaturized, and potentially low-cost solution for mid-infrared spectroscopy applications. By combining QCLs on chip, this approach addresses the limitations of conventional free-space routing systems, which are bulky, susceptible to mechanical disturbances, and require expensive optical components.

The Ge-on-Si platform, chosen for its low losses in the mid-infrared range and compatibility with CMOS processes, has been simulated to support high coupling efficiency between the QCL and the Ge waveguide. The use of Lumerical FDTD simulations guided the design to achieve an optimal coupling efficiency of 61.8%, with practical tolerances for misalignment. The use of multimode interference coupler provided broad bandwidth light combining/splitting necessary for dual-comb spectroscopy. Additionally, the process flow and fabrication methods were discussed in detail, ensuring high-quality Ge waveguides and facets.

The assembly process, including solder-based flip-chip bonding, was meticulously developed to ensure precise alignment and robust mechanical support for the QCLs. The step heights before and after solder reflow were measured to verify the correct placement of the QCLs, and the self-alignment mechanism was successfully demonstrated. Electrical connections were established through wire bonding, and the assembled device’s functionality was verified through P-I-V characterization. Post-assembly, our devices demonstrated a lasing threshold of 170 mA under pulsed operation, and stable CW operation at 15 °C with sub-milliwatt output power, suitable for sensing applications.

This work not only showcases the potential of flip-chip integration for mid-infrared photonics but also lays the groundwork for future developments in on-chip QCL systems. The successful packaging of the beam-combined chip further underscores the feasibility of miniaturizing the entire light source module, paving the way for practical industrial, environmental, and health monitoring applications. Future efforts will focus on enhancing the output power, improving thermal management, and exploring the integration of longer QCLs to expand the capabilities of the integrated mid-infrared spectroscopy systems.

## Data Availability

The data presented in this study are available upon request from the corresponding author. The simulation tool used in this study, Ansys, Inc. Lumerical FDTD, USA is commercially available via license.

## Abbreviations

The following abbreviations are used in this manuscript:

3D	three-dimensional
QCL	quantum cascade laser
PIC	photonics integrated circuit
RIE	reactive ion etching
SEM	scanning electron microscopy
AR	anti-reflection
ENIG	electroless nickel immersion gold
SEM	scanning electron microscope
LMPA	low melting point alloy
SM	single-mode
CW	continuous wave
TEC	thermoelectrical cooler
HHL	high heat load
SS	stainless steel
